# Editorial: Towards a psychophysiological approach in physical activity, exercise, and sports, volume III

**DOI:** 10.3389/fpsyg.2024.1532932

**Published:** 2024-12-19

**Authors:** Pedro Forte, José Eduardo Teixeira, Daniel Leite Portella, Diogo Monteiro

**Affiliations:** ^1^LiveWell—Research Centre for Active Living and Wellbeing, Polytechnic Institute of Bragança, Bragança, Portugal; ^2^CI-ISCE, ISCE Douro, Penafiel, Portugal; ^3^Physiotherapy and Pain Group, Department of Physical Therapy, University of Alcalá, Madrid, Spain; ^4^Department of Sports Sciences, Polytechnic Institute of Bragança, Bragança, Portugal; ^5^Department of Sports, Higher Institute of Educational Sciences of the Douro, Penafiel, Portugal; ^6^Department of Sports Sciences, Polytechnic of Guarda, Guarda, Portugal; ^7^Department of Sports Sciences, Polytechnic of Cávado and Ave, Guimarães, Portugal; ^8^SPRINT—Sport Physical Activity and Health Research and Innovation Center, Guarda, Portugal; ^9^Group of Study and Research in Physical Exercise Science, University of São Caetano do Sul, São Caetano do Sul, Brazil; ^10^Master's Programme in Innovation in Higher Education in Health, University of São Caetano do Sul, São Caetano do Sul, Brazil; ^11^Research Center in Sports, Health and Human Development, Covilhã, Portugal; ^12^ESECS-Polytechnic of Leiria, Leiria, Portugal

**Keywords:** sports, psychology, behavior, physiology, load

“*Towards a psychophysiological approach in physical activity, exercise, and sports-volume III*” explores the complex interactions between physical activity, exercise, and psychological factors in various populations and analytical contexts. A recurring theme across studies is the emphasis on how psychological factors, such as anxiety, self-confidence, and attentional control, interact with physiological outcomes like heart rate variability (HRV) and perceived exertion. Key findings include the enhancement of performance through strategic psychological interventions (e.g., mindfulness, positive thinking) and the understanding that physical activity can alleviate mental health symptoms, especially in populations with chronic conditions such as long COVID. The studies also collectively underscore the importance of a holistic approach to optimizing performance in individual and team sports, where psychological wellbeing plays a crucial role in optimizing physical outcomes. Hoffmann et al. investigated the effects of hypnosis on elite Downhill Mountain bikers. The study found that hypnosis helps athletes reduce anxiety, improve self-confidence, and enhance performance and HRV. Breido et al. developed the Sport Preference Questionnaire (SPOQ) to assess the psychological effects of physical activity in children with mental illness. Results showed that different psychiatric conditions influenced physical activity levels and perceptions of fitness. Wu et al. explored RPE-derived metrics for predicting injury risk in curlers and found that the Exponentially Weighted Moving Average (EWMA) metric without delay is the most accurate variable. Moura et al. examined the relationship between psychological factors and performance in long and triple jumpers, finding that emotion regulation and self-control were key to better performance. A study by Liu et al. highlighted the impact of expressive ties on competitive performance using Dance Sport dyads, showing that emotional intelligence (EI) influences performance through athlete engagement. Wang et al. investigated the relationship between coaching behavior, team cohesion, and competitive anxiety in handball, suggesting that fostering cohesion and promoting task-oriented goals can alleviate anxiety. Qi and Jinmin used Mendelian randomization to show that cognitive performance mediates the relationship between education and physical activity. Schittenhelm et al. compared slow- vs. fast-beat music during rowing, finding that while fast music improved performance, slower-tempo music is adequate for recovery. Grønset et al. examined how mental processes like arousal regulation and mental toughness affect performance in football and found that mental toughness plays a critical role in overcoming challenges. Shi et al. found that mindfulness training improved attentional control during football penalty shootouts, highlighting its potential to reduce anxiety and enhance performance. Ferreira et al. assessed Project SCORE in promoting Positive Youth Development, showing improvements in athletes' life skills. Teixeira et al. used machine learning to predict recovery states in youth football players, achieving high classification accuracy. Yu et al. reviewed exercise interventions for maternal depression, anxiety, and fatigue, showing that yoga and Pilates were most effective for specific symptoms. A study by Gao et al. on sport and personality in adolescents showed that different activities influenced traits like openness and conscientiousness, with significant differences between genders. Rawls and Finomore examined attentional focus during perceptual tasks, finding that external focus reduces internal workload and stress. Sirotiak et al. investigated the impact of physical activity on health in individuals with long COVID and found that higher levels of physical activity improved perceived health outcomes. Sim et al. studied sport anxiety and life satisfaction in male athletes, noting that positive thinking skills moderate the relationship between anxiety and satisfaction. Tolukan et al. analyzed error perception in athletes with disabilities, highlighting the role of reflection and challenge in deliberate practice.

The main conclusions drawn from these studies highlight the critical role of psychological factors in enhancing physical performance, particularly in the context of sports and physical activity. Interventions aimed at improving psychological resilience, such as mindfulness, positive thinking, and anxiety management, have been shown to be effective in boosting athletes' performance and overall wellbeing. In the context of physical activity, exercise, and sport prescribing, these findings suggest that incorporating psychological training into exercise regimens can be highly beneficial, particularly for athletes facing high levels of stress or chronic health conditions. A deeper understanding of the dynamic interaction between the mind and body will be possible through the incorporation of these technologies into research approaches. Research is using neuroimaging to map patterns of brain activity linked to motivation, focus, judgement, and maximizing performance. In the coming years, researchers will investigate novel psychophysiological interventions aimed at enhancing performance and boosting health in a variety of populations, building on current knowledge. All of these assumptions have been and will continue to be areas of interest to explore for a psychophysiological approach to physical activity, exercise and sport.

